# Supplementary material for: Examining the Relationships between the Incidence of Infectious Diseases and Mood Disorders: An Analysis of Data from the Global Burden of Disease Studies, 1990–2019
Source: Diseases. 2023 Sep 6;11(3):116. doi: 10.3390/diseases11030116 (PMC10528187; doi:10.3390/diseases11030116)
Supplement: Supplementary file 1 [file diseases-11-00116-s001.zip › Table S1.docx]

**Supplementary Table S1: Correlations involving possible confounding or interacting variables**

**Table S1a: Correlations between the incidence of mood disorders and possible confounders**

| **Variable** | **Distance from the equator** | **Human Development Index** | **Gini coefficient** | **Urbanization  (% living in cities)** |
| --- | --- | --- | --- | --- |
| **MDD, incidence**  1990  2019 | .16 (.025)*  .07 (.313) | -.17 (.053)  -.24 (.008)* | .08 (.333)  .19 (.015)* | .04 (.614)  .02 (.795) |
| **BD, incidence**  1990  2019 | .14 (.043)* .15 (.032)* | .43 (<.001)**  .34 (<.001)** | .12 (.110)  .18 (.022)* | .43 (<.001)**  .38 (<.001)** |

**Table S1b: Correlations between the incidence of infectious diseases and possible confounders**

| **Variable** | **Distance from the equator** | **Human Development Index** | **Gini coefficient** | **Urbanization  (% living in cities)** |
| --- | --- | --- | --- | --- |
| **Upper respiratory infections**  1990  2019 | .21 (.003)*  .25 (.001)* | .76 (<.001)**  .67 (<.001)** | -.21 (.006)*  -.11 (.173) | .59 (<.001)**  .55 (<.001)** |
| **Lower respiratory infections**  1990  2019 | -.51 (<.001)**  -.55 (<.001)** | -.83 (<.001)**  -.81 (<.001)** | .44 (<.001)**  .42 (<.001)** | -.65 (<.001)**  -.56 (<.001)** |
| **Enteric infections**  1990  2019 | -.28 (<.001)**  -.36 (<.001)** | -.75 (<.001)**  -.74 (<.001)** | .25 (<.001)**  .18 (.018)* | -.59 (<.001)**  -.51 (<.001)** |
| **Tropical infections**  1990  2019 | -.69 (<.001)**  -.70 (<.001)** | -.85 (<.001)**  -.83 (<.001)** | .54 (<.001)**  .50 (<.001)** | -.68 (<.001)**  -.54 (<.001)** |
| **Other infectious diseases**  1990  2019 | -.56 (<.001)**  -.57 (<.001)** | -.92 (<.001)**  -.89 (<.001)** | .44 (<.001)**  .36 (<.001)** | -.73 (<.001)**  -.64 (<.001)** |

**Note:** All correlations are given as Spearman’s ρ (*p-*value). All variables in the first columns refer to incidence estimates.

**Abbreviations:** MDD, major depressive disorder; BD, bipolar disorder.

***** Significant at *p* < 0.05.

** Significant at *p* < 0.05 after Bonferroni correction for multiple comparisons.
