# Supplementary material for: Examining the Relationships between the Incidence of Infectious Diseases and Mood Disorders: An Analysis of Data from the Global Burden of Disease Studies, 1990–2019
Source: Diseases. 2023 Sep 6;11(3):116. doi: 10.3390/diseases11030116 (PMC10528187; doi:10.3390/diseases11030116)
Supplement: Supplementary file 1 [file diseases-11-00116-s001.zip › Table S2.docx]

**Supplementary Table S2: Inter-correlations between the incidence of distinct categories of infectious disease**

**Table S2a: Correlations for the year 1990**

| **Disease category** | **1**  **URI** | **2**  **LRI** | **3**  **Enteric** | **4**  **Tropical** | **6**  **Other** |
| --- | --- | --- | --- | --- | --- |
| **1** | - | -.64 (<.001)* | -.91 (<.001)* | -.54 (<.001)* | -.65 (<.001)* |
| **2** |  | - | .61 (<.001)* | .74 (<.001)* | .80 (<.001)* |
| **3** |  |  | - | .54 (<.001)* | .64 (<.001)* |
| **4** |  |  |  | .78 (<.001)* | .77 (<.001)* |
| **5** |  |  |  | - | .83 (<.001)* |

**Table S2b: Correlations for the year 2019**

| **Disease category** | **1**  **URI** | **2**  **LRI** | **3**  **Enteric** | **4**  **Tropical** | **5**  **Other** |
| --- | --- | --- | --- | --- | --- |
| **2** | - | -.66 (<.001)* | -.90 (<.001)* | -.53 (<.001)* | -.72 (<.001)* |
| **3** |  | - | .68 (<.001)* | .76 (<.001)* | .78 (<.001)* |
| **4** |  |  | - | .61 (<.001)* | .78 (<.001)* |
| **5** |  |  |  | .78 (<.001)* | .74 (<.001)* |

**Note:** All correlations are presented as Spearman’s ρ (*p-*value). All variables refer to incidence estimates.

**Abbreviations:** URI, upper respiratory infections; LRI, lower respiratory infections.

* Significant at *p* < 0.05 after Bonferroni correction for multiple comparisons.
