# Supplementary material for: Examining the Relationships between the Incidence of Infectious Diseases and Mood Disorders: An Analysis of Data from the Global Burden of Disease Studies, 1990–2019
Source: Diseases. 2023 Sep 6;11(3):116. doi: 10.3390/diseases11030116 (PMC10528187; doi:10.3390/diseases11030116)
Supplement: Supplementary file 1 [file diseases-11-00116-s001.zip › Table S3.docx]

**Supplementary Table S3: Correlations between the incidence of each category of infectious disease and mood disorders, adjusted for other categories of infectious disease**

**Table S3a: Cross-sectional correlations**

| **Correlation** | **1990** | **2019** |
| --- | --- | --- |
| **Upper respiratory infections**  **x Major depressive disorder x Bipolar disorder** | -.11 (.108)  .45 (<.001)** | .08 (.241)  .01 (.877) |
| **Lower respiratory infections**  **x Major depressive disorder x Bipolar disorder** | .12 (.107)  .11 (.126) | .12 (.101)  .05 (.520) |
| **Enteric infections**  **x Major depressive disorder x Bipolar disorder** | -.16 (.026)*  -.25 (<.001)* | .04 (.554)  -.15 (.039)* |
| **Tropical infections**  **x Major depressive disorder x Bipolar disorder** | -.09 (.226)  .18 (.013)* | -.12 (.085)  -.05 (.518) |
| **Other infectious diseases**  **x Major depressive disorder x Bipolar disorder** | .05 (.480)  -.27 (<.001)** | .09 (.231)  -.05 (.496) |

**Table S3b: Correlations between percentage changes**

| **Disease category** | **Major depressive disorder** | **Bipolar disorder** |
| --- | --- | --- |
| **Upper respiratory infections** | -.07 (.352) | .66 (<.001)** |
| **Lower respiratory infections** | .29 (<.001)** | -.01 (.905) |
| **Enteric infections** | -.14 (.047)* | -.23 (.001)* |
| **Tropical infections** | -.21 (.003)* | -.14 (.046)* |
| **Other infectious diseases** | -.09 (.209)* | -.02 (.773) |

**Note:** All correlations are presented as Spearman’s partial ρ (*p*-value). All partial correlations are adjusted for the other categories of infectious disease (e.g., the correlation “upper respiratory infections x major depressive disorder” is adjusted for lower respiratory, enteric, tropical, and other infections).
* *p* < .05, uncorrected.
** *p* < .05 after Bonferroni correction for multiple comparisons.
