# Supplementary figures and images for: Examining the Relationships between the Incidence of Infectious Diseases and Mood Disorders: An Analysis of Data from the Global Burden of Disease Studies, 1990–2019
Source: Diseases. 2023 Sep 6;11(3):116. doi: 10.3390/diseases11030116 (PMC10528187; doi:10.3390/diseases11030116)

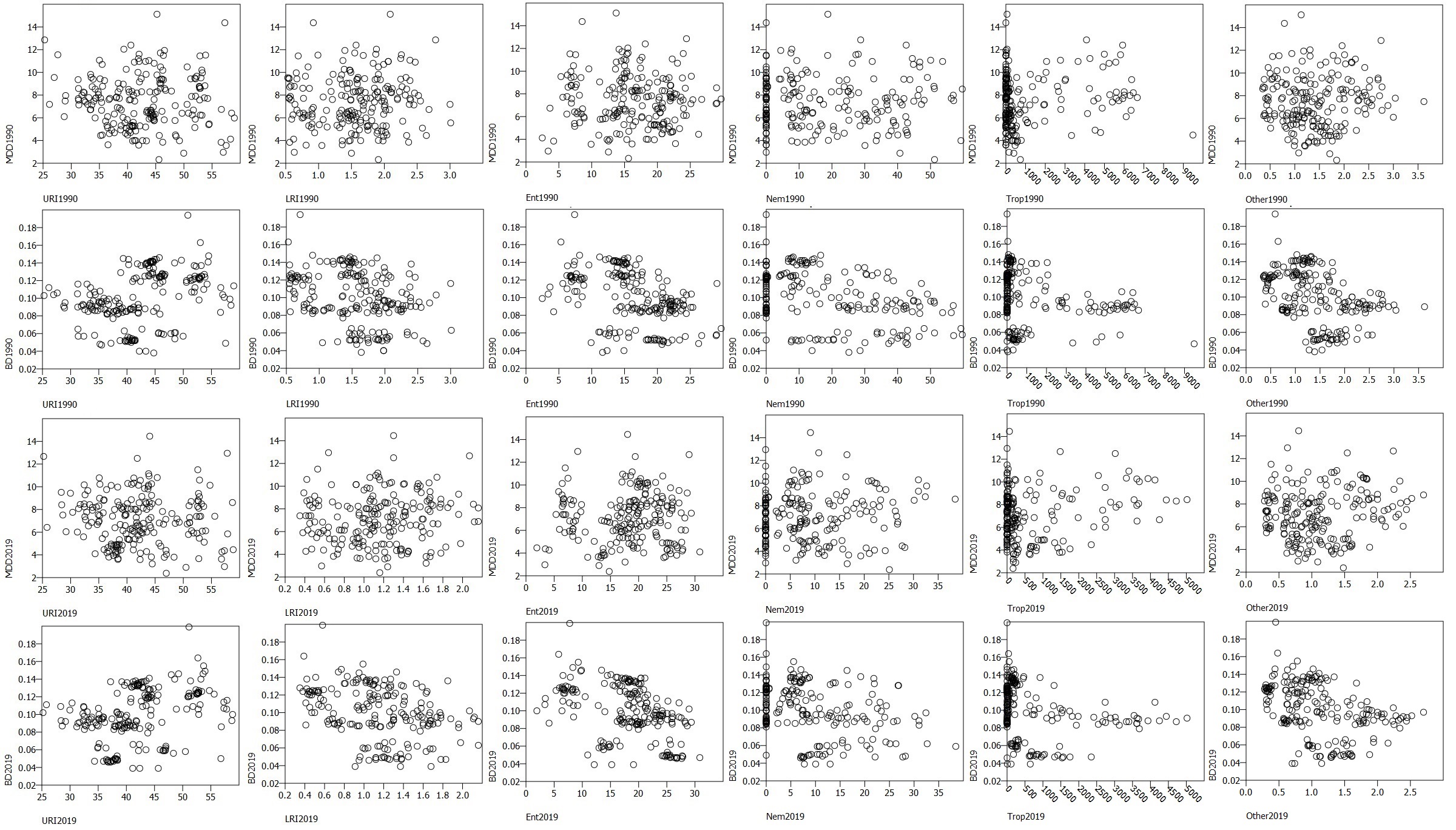

Supplement: Supplementary file 1 [file diseases-11-00116-s001.zip › Figure S1.jpg]
